# Understanding Human-*Plasmodium falciparum* Immune Interactions Uncovers the Immunological Role of Worms

**DOI:** 10.1371/journal.pone.0009309

**Published:** 2010-02-19

**Authors:** Christian Roussilhon, Philippe Brasseur, Patrice Agnamey, Jean-Louis Pérignon, Pierre Druilhe

**Affiliations:** 1 Unité de Parasitologie Bio-Médicale, Institut Pasteur, Paris, France; 2 UMR 198, Institut de Recherche pour le Développement, Dakar, Sénégal; 3 Laboratoire de Parasitologie-Mycologie, Hopital Sud, CHU d'Amiens, Amiens, France; Queensland Institute of Medical Research, Australia

## Abstract

**Background:**

Former studies have pointed to a monocyte-dependant effect of antibodies in protection against malaria and thereby to cytophilic antibodies IgG1 and IgG3, which trigger monocyte receptors. Field investigations have further documented that a switch from non-cytophilic to cytophilic classes of antimalarial antibodies was associated with protection. The hypothesis that the non-cytophilic isotype imbalance could be related to concomittant helminthic infections was supported by several interventions and case-control studies.

**Methods and Findings:**

We investigated here the hypothesis that the delayed acquisition of immunity to malaria could be related to a worm-induced Th2 drive on antimalarial immune responses. IgG1 to IgG4 responses against 6 different parasite-derived antigens were analyzed in sera from 203 Senegalese children, half carrying intestinal worms, presenting 421 clinical malaria attacks over 51 months. Results show a significant correlation between the occurrence of malaria attacks, worm carriage (particularly that of hookworms) and a decrease in cytophilic IgG1 and IgG3 responses and an increase in non-cytophilic IgG4 response to the merozoite stage protein 3 (MSP3) vaccine candidate.

**Conclusion:**

The results confirm the association with protection of anti-MSP3 cytophilic responses, confirm in one additional setting that worms increase malaria morbidity and show a Th2 worm-driven pattern of anti-malarial immune responses. They document why large anthelminthic mass treatments may be worth being assessed as malaria control policies.

## Introduction

Deciphering the interactions between the malaria parasite with the human host and understanding how we acquire immunity to malaria has been fundamental to our malaria vaccine research program. Over the course of the program, we have extensively studied the characteristics of immune responses of children and adults to malaria [1]. In particular, the distribution of IgG antibody subclasses has led us to suspect the role of helminthic infections on acquisition of immunity to malaria [2]. A cascade of observations led progressively over time to corroborate this suspicion.

The pattern of acquisition of resistance to malaria and the role of antibodies should be reminded first. Children in hyperendemic areas are at high risk of dying from malaria until the age of 5. Though this risk then decreases, they remain susceptible to malaria attacks until they reach the ages of 15–20 years [3]. By the time they are young adults, those who have survived achieve a remarkable state of premunition where they are able to control parasite growth below the threshold at which clinical symptoms occur [4]. They have acquired immunity, but at remarkably low speed. It has been conceptually difficult for a long time to understand the reason for this long delay: the question being how malaria antigens could be so poorly immunogenic that daily exposure to outstandingly high parasite loads for many years would be required before individuals are protected against the disease.

This immunity is due to antibodies. Protection can be induced by passive transfer of IgG from malaria-immune adults to malaria patients [5]. In order to understand the underlying mechanism we compared antibody responses between those protected and those not protected [6]. We found this protection to be associated with a novel immune mechanism we called “Antibody Dependant Cellular Inhibition (ADCI)” in which effective antibodies act in a monocyte (MN)-mediated manner [7]. The ADCI assay measuring this potential protective immune mechanism has been used to screen a genome-wide expression library in which MSP3 was identified as the main target of antibodies mediating ADCI [8]. GLURP (Glutamate-rich Protein) and SERP (Serine-stretch or Serine-rich Protein) were later identified as additional targets [9], [10]. In our efforts to better characterize the molecular events leading to ADCI activity, we observed that only minute amounts of antibodies in the range at which hormones act, were required [11]. We further found that, in ADCI, an essential component of the MN mediated antiparasitc effect is the synergistic activation of monocyte receptors Fcγ RIIa and Fcγ RIIIa by cytophilic classes of IgG bound to at least a bivalent antigen [11]. This evidence of the essential trigerring role of cytophilic classes of antibodies, namely IgG1 and /or IgG3, the only ones able to bind receptors and activate monocytes, led us subsequently to investigate the isotype distribution of antimalarial antibodies [12].

These studies, the first of their kind, showed that, in contrast to current beliefs, immune responses were not absent in non-protected individuals. In fact, they were present and abundant, but were qualitatively different. Among non-protected individuals, aged 1–10 and up to 15 years, non-cytophilic classes of antibodies IgG2, IgG4, and IgM were the most abundant. This stood in stark contrast to responses in protected adults who had twice as much cytophilic IgG1 and IgG3 sub-classes compared to the noncytophilic classes [12]. In other words the balance of antibodies is more critical for protection than their abundance. This indicated that those protected individuals had acquired the ability for an IgG class-switch from a predominantly non-cytophilic to a cytophilic predominance. Further studies pin-pointed IgG3 against MSP3 as the antibody response most strongly associated with protection [1]. The identification of an IgG class-switch associated with the acquisition of protection, also provided, for the first time, a plausible explanation for the abnormally long delay needed to acquire immune protection. The long delay was not due to the need to accumulate high titers of antibodies to every antigen, but that required to achieving an Ig class switch. Simultaneously, this finding provided a biological target for developing a malaria vaccine, the induction of cytophilic antibodies in young children.

This, in turn raised the question of why children and adolescents were producing a majority of non-cytophilic classes of antibodies. The possibility that this was merely due to an immaturity of the immune system during childhood and adolescence seemed unlikely. We considered the fact that, children often differ from adults in malaria endemic areas by the outstandingly high loads of worms they harbour [13]. Indeed, high helminthic loads are known to be associated with a Th2/Th1 imbalance [14] and therefore, could be responsible for a worm-induced Th2 cytokines-mediated drive upon immune responses directed to malarial antigens, over childhood and adolescence.

To explore the possibility that helminthic infections may have an effect on acquisition of clinical immunity against malaria, we initiated several studies on worm-malaria interactions. Most of them indicated a deleterious role of worms on malaria immunity [2]. In an intervention study in Ankazobe (Madagascar) which compared malaria attacks occurring in children 2–15 years of age, harbouring intestinal worms, who were randomly assigned to receive either the anthelminthic levamisole or a placebo, a 2.5 fold decrease in the risk of malaria attacks occurred in the worms-treated group. Noticeably, this effect became apparent only 18–24 months after worm treatment, which was expected as it implied the induction of an Ig class switch in B-lymphocytes [2].

Numerous case control studies that compared worm-infected with worm-free individuals (without anthelminthic treatment), confirmed a markedly higher incidence of clinical malaria in wormy individuals and showed that all types of helminths could be involved, either intestinal parasites, such as *Ancylostoma*, *Ascaris*, *Trichuris*, *Enterobius*, or systemic parasites such as *Schistosoma mansonii* and *haematobium* (reviewed in [2], [15], [16], [17]). However, there exists also studies that have observed the opposite [2], [15], [16], [17]. We feel that most of these studies failed to include the assessment of the load of worm. In a study we conducted in Northern Senegal to look into the interactions between Schistosomiasis and malaria, we observed that malaria incidence was increased at high and medium *Schistosoma* egg-load, whereas at very low egg-loads, a trend towards the opposite effect was seen [18]. Therefore, when the other studies have found that adults with worms are still protected, we contend that this corresponds mostly to adults with comparatively lower worm loads who have achieved clinical immunity anyway. In support of this view, most studies of experimental co-infections in models, in which the worm load is usually high since it is an induced infection, concluded to their deleterious influence upon malaria [19], [20], [21], [22], with a single exception in a rodent model of cerebral malaria [23].

Although the results from the clinical studies indicated a clear interaction between worm infestations and occurrence of malaria attacks, they could not address the immunological hypothesis of a Thelper type 2 imbalance due to worms, either because the groups were too small to provide significant immunological data or simply because no biological specimens were available in those retrospective studies.

We therefore undertook a prospective study in Casamance, Southern Senegal, in a cohort of 203 children who were assessed at the beginning of the study during the non-transmission season, and closely followed-up for a total of 51 months. Immunological, epidemiological and clinical data gathered in this manner provide, for the first time, the grounds to our immunological hypothesis of a worm-induced Th2 drive.

## Materials and Methods

### Study Design

The study was designed to be a prospective study aimed at following a cohort of children 1–14 years of age (mean±1SD: 6.9±3.1) for the occurrence of worms and attacks of malaria over a 4 year period (51 months).

### Study Site

It took place in Mlomp, a village of ca. 8,000 inhabitants in Casamance, South West Senegal, where the local economy is based on subsistence farming, rice culture and fishing. Three similar adjoining suburbs surrounding the local Health care Unit were selected. All households are built on an identical model (houses are built with earth-bricks locally produced and are covered with corrugated iron) and scattered in a similarly forested environment characterized by a few high trees. The village is surrounded by numerous rice fields which are a major source of intestinal helminth infections. At the start of the survey, bednets were not used by the children, and this was frequently verified throughout the 51 month follow-up.

Malaria transmission is seasonal, with a low monthly incidence of malaria attacks in June, which gradually increases up to a maximum in September, approximately 3 months after the peak of rainfalls. During the study period, the incidence of malaria morbididy was 52% and *Plasmodium (P). falciparum* was present in more than 95% of the clinical malaria cases. The main vector was *Anopheles gambiae* with an estimated 25 infective bites per individual per year in this village.

### Inclusion Criteria

This longitudinal study took place between November 2000 and January 2005. The cohort was constituded based on an initial stool examination of 455 children. In order to constitute two sub-groups of equal size, one with, the other without worms, and paired for age, i.e. with no age imbalance between the two groups, 238 out of the 455 children aged 1–14 years could be included in the study according to those criteria. Thirty-five children could not be followed-up continuously at each time point, and therefore, only 203 (85.3%) children fulfilled the requirement of continuous follow-up during the 51 months of survey and were included in the final analysis

This study received ethical approval from the National Ethical Committee of Senegal. All patients (or their parents) gave formal informed consent after detailed explanations given in Diola language by a local health care assistant and the agreement was systematically obtained in the presence of a witness from the village.

### Stool Examinations

Stool samples were obtained at enrolment and annually from each child and screened for eggs and parasites by trained parasitologists both by microscopic observation of stools, as well as after using the eggs concentration technique described by Bailenger [24].

Briefly, stools were diluted 10 times in a dilution buffer (Na acetate 15g, acetic acid 3,60 ml, in 1000 ml water adjusted at pH5). After 1 minute of incubation, the stools suspension was filtered through a metalic mesh and a volume of sulfuric ether was added to the same volume of filtered material in a centrifuge tube, and then emulsified by vigorous agitation. Following centrifugation at 1500 rpm for 1 minute, the pellet was sampled for microscopic examination. Stools found negative were re-tested a second time, a few days later.

In Mlomp village, the parasite loads, mostly due to hookworms, reached an average of 1526 eggs/gramme of faeces, i.e. can be considered moderate to high.

Stool examinations were repeated every year and individuals from the negative group who had become positive on year 1 and 2 were treated with 400 mg Albendazol®. In addition, it was agreed that, if clinical symptoms associated with helminth infection would occur, the child would be treated of his parasite infection by the nurse running the local dispensary.

Finally, on year 3 and 4, in agreement with our initial protocol and in order to comply with ethical considerations, an anti-helminthic treatment of 400 mg Albendazol® was given once a year to all participants.

### Identification of Clinical Malaria Attacks

The incidence of malaria attacks was measured by both active and passive case detection. Parents were asked to refer children to the dispensary in case of any abnormal symptom. In addition, households were visited at least weekly by the medical team continuously stationed in the village during the transmission season, so as to ascertain that suspected cases had been referred to the dispensary. Compliance estimated by weekly visits was high, particularly as nurses from the dispensary deliver free medical care. For each single case of fever (i.e. axillary temperature ≧37.5°C), a complete clinical examination was performed by a trained medical staff and under the supervision of the principal investigator (Dr. P.B.). A thick blood smear was systematically made and rapidly examined for the presence of malaria parasites. Slides were stained with Giemsa and examined against 200 leukocytes if positive, or against 400 leukocytes prior to being declared negative.

A *P. falciparum* malaria attack was defined by the presence of both fever and *P. falciparum* parasites with no clinically apparent other cause of fever. Children presenting with a malaria attack were given an antimalarial curative treatment. Intramuscular quinine injection was given in 95% of the clinical cases, followed by oral quinine for 4–5 days (25mg/kg/day in three doses). Over the two last years of the survey quinine was progressively replaced by artesunate-amodiaquine combination (arsumax™ and camoquin™), which accounted the last year for about 50% of the treatments. Recovery was considered as a further indication that the febrile episode was indeed an actual clinical malaria attack. From these data, prevalence, incidence density per time unit (month, 6 months, year), and geometric mean annual incidence, used to express the risk of malaria in the study population were calculated.

No case of complicated malaria was recorded in the cohort of children enrolled for this study during the entire follow-up.

### Sera Samples and ELISA Assays

Sera samples were obtained by venous puncture at the start of the survey (in November 2000) from each child of the cohort and the parasite-specific antibody contents from this first year blood samplings were evaluated by ELISA. Individual sera were tested for IgG1, IgG2, IgG3 and IgG4 subclasses against various *P. falciparum* antigens.

The *P. falciparum*-derived antigens (amino-acid numbers referring to 3D7 clone) tested were *a*) MSP3b_(184–210)_ a 27 AA long synthetic peptide corresponding to the major B cell epitope of MSP3 [25], b) MSP3-DG210_(163–230)_, including the same region c) MSP3-CTerminus_(191–354)_, AMA1 a gift of Dr Robin Anders, GLURP-R0, a kind gift of M. Theisen [9], and SERP, a kind gift of Dr. T. Horii [26], the later 5 corresponding to recombinant proteins.

IgG subclass antibodies to the different antigens were detected and quantified essentially as previously described [7], [27], [28]. Briefly, high-binding 96-well microplates (Nunc Maxisorp®, Denmark) were coated with 5µg of antigen per well. Antigens were dissolved in PBS and incubated overnight at 4°C. The optimal serum dilution was determined to be 1∶200 except for MSP3b, which was tested at 1∶50 dilution, as in our previous studies [1], [25], [29].

Human subclass specific mouse monoclonal Abs were used and assayed using peroxidase-labelled goat anti-mouse IgG (at 1∶2000) as previously described [7], [27], [28]. Optimal concentrations for the selected reagents were 1∶2000 for IgG1 (clone NL16), 1∶10,000 for IgG2 (clone HP6002) and IgG3 (clone ZG4), and 1∶30,000 for IgG4 (clone RJ4 from Skybio, UK). Peroxidase-conjugated polyclonal goat anti-human IgG (γ-chain specific) were diluted 1∶10,000.

Antibody responses are expressed as Arbitrary Units obtained by calculating the ratio of test OD values obtained for a given test sample divided by a background value defined as the mean plus 3 SD of the absorbance values obtained using a subsample of 40 negative control sera from malaria-naïve blood donors.

### Statistical Analysis of Data

We analyzed the data using the JMP® software and Statview® 5 softwares, both from SAS Institute (Cary, North Carolina, United States). We used univariate and multivariate analysis to explore factors potentially associated with susceptibility to malaria attacks. *P. falciparum*-specific humoral responses were tested by two successive approaches. Firstly, the pattern of parasite-specific antibody responses was evaluated with regard to the presence or absence of intestinal helminths (used as a dichotomous variable) in univariate tests. Then, age and parasite-specific antibody responses were used as continuous explanatory variables in nominal logistic regressions. Secondly, the pattern of *P. falciparum*-specific humoral responses was determined with regard to the occurence or non-occurence of malaria attacks during the 51 months of follow-up. In additional studies, the (Log+1)-transformed total number of clinical episodes was used as a continuous variable and the explanatory variables included in multivariate analysis were age sex, household/family and the antibody responses, expressed as Log-transformed arbitrary units as in previous studies [1], [25], [29].

We tested the hypothesis that a) children with high levels of antibodies to some of the parasite antigens tested were less susceptible to clinical malaria and b) children without worms differed from children with helminths in term of relative susceptibility to malaria. The different helminth infections observed were initially grouped in one single binary exposure variable corresponding to the presence or absence of helminth infection. We also looked for a potential individual effect of some specific type of infections after identification of the infecting parasite species. We first looked at the 2 subgroups separately using non-parametric tests to analyze non-Gaussian variables, whereas variables with normal distribution were tested by parametric tests. Multiple linear regression models were used to determine the potential effect of different explanatory variables (such as age and *P. falciparum*-specific antibody responses) on the significance of the comparisons. A stepwise logistic regression model retaining explanatory variables with *P* values less than 0.05 in the initial study was used to reduce the number of explanatory variables tested in the full model.

## Results

### Incidence of Malaria Attacks and Prevalence of Intestinal Helminth Infections in the Study Population

The 203 children from Mlomp had a mean age of 6.9±3.1 years (range: 1–14 years) at enrolment in the year 2000. At baseline, children without intestinal helminths (n = 98) were 6.8±3.4 years old (range: 1–13 years) whereas children with worm infections (n = 105) were 7.0±2.9 years old (range: 1–14 years), i.e. the 2 subgroups of children did not differ for age. Three years after the start of the follow-up, the weight of the children was comparable between the 2 subgroups (26.5±9.7 kg versus 28.7±11.3 kg, in the group without and with intestinal helminths respectively). The sex ratio was similar in the two subgroups with 46.8% males.

Over the duration of the study, the prevalence of malaria attacks peaked three months after the end of the rainy season (not shown). The monthly prevalence of symptomatic malaria episodes ranged from 0% in May 2001 up to 13% in November of the same year. The geometric mean annual incidence *rate* of malaria attacks per child was 0.614 (95% CI = 0.503–0.734).

During the 51 months period of active and continuous survey, 47 out of 203 children (23.1%) had no detectable episode of symptomatic malaria diagnosed, whereas 421 malaria attacks were recorded in the 156 children who experienced between 1 to 9 clinical malaria episodes (mean ± 1SD = 2.7±1.7 attacks/child), and received first-line treatments. Fourty five children had 1 attack, 42 had 2 attacks, 29 had 3, and 40 had >4 attacks (range 4–9 attacks). The incidence of malaria episodes over the study period ranged from 81.13% (2.43±2.10 attacks/51 months) in 53 children aged 0–4 years, to 72.34% (1.81±1.85 attacks/51 months) in 47 children aged 10–14 years.

During the study, and notably during the last two years of the survey, the occurrence of clinical malaria in the cohort of children was found to progressively decrease with time (Rho = −0.44; *P* = 0.0014). This was most likely due to a combination of factors including improved access to medical treatment, introduction of artesunate-amodiaquine combination [30], and the increase in age of the children enrolled in the study (Rho = −0.127; *P* = 0.069).

105 out of 203 children (51.7%) were found infected at enrollment and with at least one intestinal helminth species. 60% of the remaining 98 children initially free of detectable helminthic infection remained so throughout the study. Fourty percent of helminth free children became helminth positive over the follow-up period (3.1% after 1 year, 8.2% after 2 years, 13.3% after 3 years, 15.3% at 51 months), 14 being infected twice.

The most frequent species was hookworm, detected in 87 of the helminth-infected children. *Ascaris lumbricoides* was found in 21, *Strongyloïdes stercoralis* in 17, *Enterobius vermicularis* and *Trichuris trichiura* in 6, and *Hymenolepis nana* in 3 individuals. Prevalence of each helminth species did not differ significantly depending on gender, age, or year of study. A mean number of 1526 eggs per gramme of stools (range: 40–5800) was determined by the concentration technique employed, and only 10% of the children with helminth infections had less than 80 eggs per g of stools, indicating moderate to high helminthic loads in this cohort of children.

We addressed the existence of potential differences in spatial distribution of the prevalence of infections either as a function of neighbourhood, of household or of family links. Children were living in 3 main and geographically contiguous areas of Mlomp and belonged to 6 major family groups living in compounds similarly structured. As far as this could be evaluated, the everyday way of life, the social status, the housings and the socioeconomic indicators were very similar from one family to the other, with most parents working as agricultors in rice fields surrounding the village. On the basis of the above parameters, the prevalence of intestinal helminths, the occurrence of malaria attacks and/or the delay observed before occurrence of the first malaria attack, did not show any particular geographic clustering, i.e. these factors were equally distributed in the different geographic locations of the children. Of note, in several occasions, both helminth infected and non-infected children were identified in a same family.

### The Incidence of Malaria Attacks Is Increased in Helminth-Infected Children

The results show that the presence of any helminth species was significantly associated with an increased risk of malaria attack (Table 1). During the first year of survey, the incidence density of malaria attacks was 0.655/child/year when children had intestinal helminths, whereas it was 0.380/child/year when children were free of intestinal helminths. The ratio of malaria attack incidence density between the 2 groups of children was 1.723 (*P* = 0.0215). Over the first two years of follow-up, children with intestinal helminths had 1.943±0.309 malaria attacks, whereas children without helminths had 1.187±0.107 attacks (*P* = 0.0057).

**Table 1:** Relative risk of malaria attacks in children living in Mlomp village.

RR at 6 months =	1.60 [1.03–2.45]	*P* = 0.021
RR at 12 months =	1.72 [1.19–2.42]	*P* = 0.021
RR at 24 months =	1.53 [1.11–2.40];	*P* = 0.006
RR at 51 months =	1.24 [1.06–1.43]	*P* = 0.004

The relative risk (RR) and [95% Confidence Interval] of malaria attacks was determined in children with helminth infections compared to children without helminth infections.

When comparing 2 similar transmission periods of 6 months at 1 year interval, i.e. in 2000 and 2001, the likelihood of developing symptomatic malaria was markedly higher for children with intestinal helminths than for those without detectable helminth infection (Odds ratio = OR = 2.27; 95%CI = [1.12–4.61]; *P* = 0.0218).

After 51 months of follow up, the higher incidence of malaria attacks remained in helminth-positive as compared to helminth-free individuals (χ^2^ with Yates'correction: 7.32; *P* = 0.0068). OR and 95%CI were 2.72 (1.35–5.45).

At the end of the survey, Kaplan Meier estimates showed that 28.7% of worm free children had not suffered from clinical malaria, whereas only 11.7% of helminth positive children, i.e. 2.45 fold less individuals, remained free of symptomatic malaria, (Log Rank χ^2^ = 10.67; *P* = 0.0011).

We further studied this association in a multivariate analysis using gender, age, presence or absence of intestinal helminths as explanatory variables. The odds ratios for the occurrence of at least one episode of clinical malaria were determined after adjusting for age, sex, and household/family.

All the available variables with a potential impact on malaria attacks were included in a logistic-regression model. Stepwise logistic regression analysis starting with a full model retained helminth infection [odds ratio = 2.69; and 95% confidence interval CI = 1.34–5.39; *P* = 0.0045] as the most significant explanatory variable for an increased risk of malaria attacks whereas age was weakly associated with a decreased risk of clinical episode (odds ratio: 0.28, 95%CI = 0.06–1.20; *P* = 0.0907).

Thus the association between helminth infections and risk of malaria attacks remained significant after controlling for potential confounders. Given that all the children of the cohort lived in a similar environment and that anti-parasite immune responses were comparable between the children originating from the different compounds of Mlomp village (see below), it was considered that the risk of exposure to malaria was similar for all children.

Therefore, in the present study we conclude that the major risk factor associated with clinical episodes of malaria was whether or not the children had detectable intestinal helminthic infection(s) at the beginning of the 51 months of follow-up

### Cytophilic Anti-MSP3 Antibodies Are Associated with a Reduced Risk of Malaria Attack

Antibodies specific for each malaria antigen tested were detected in the children. For total IgG, there was no statistically significant difference in the magnitude of the responses to any antigen between the two groups, although responses tended to be higher in the “protected” group (i.e. without malaria attacks) than in the “susceptible” one (i.e. with malaria attacks). Futher studies analyzed isotype specific responses to each antigen.

**Figure 1:**
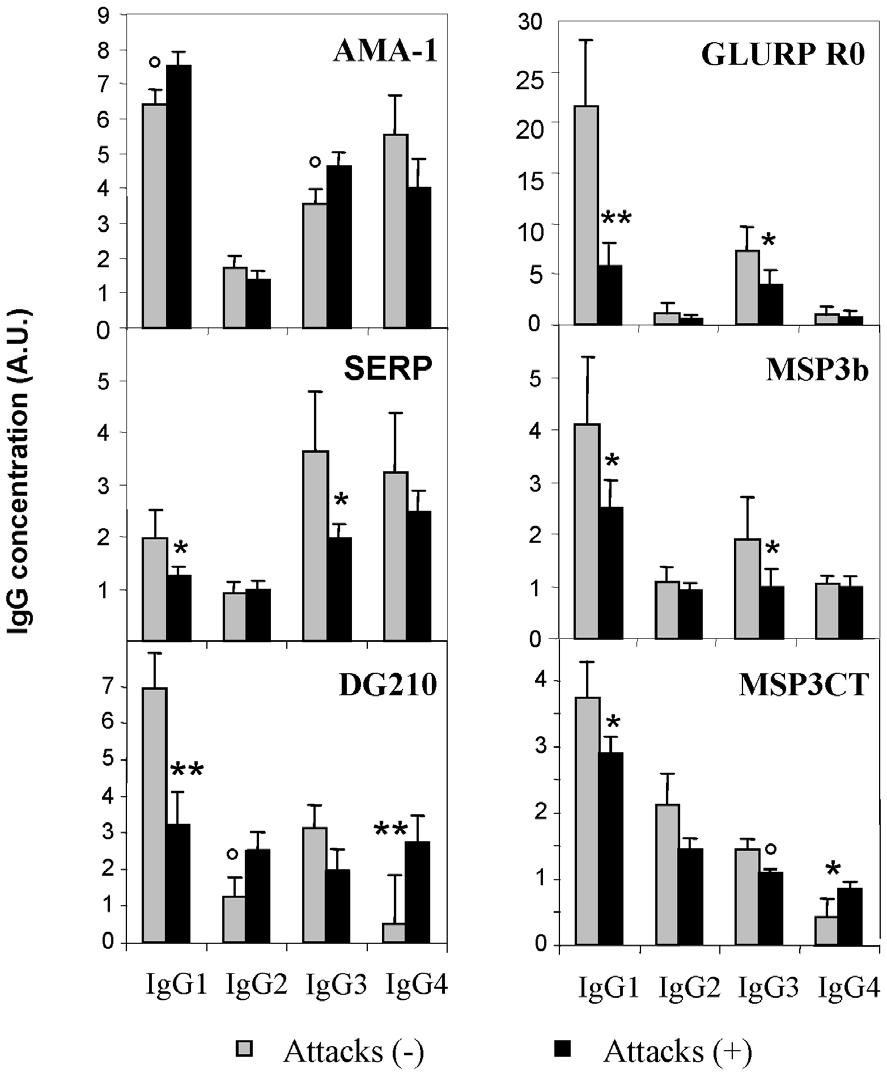
Antigen-specific antibody responses and occurence of malaria attacks. Mean reactivity in ELISA assays determined in sera from children presenting with (malaria attacks+), or without (malaria attacks−) clinical malaria during the follow-up. Sera samples were tested using different antigens and mean results for AMA-1, SERP-SERA, GLURP-R0, MSP3b, DG210 and MSP3CT are shown with standard errors of the mean IgG A.U. Labels indicate when differences were found significant in univariate analysis: ° when 0.06>p≥0.05, * when 0.05<p ≥0.0021 and ** when p<0.0021 (a p value of 0.0021 accounts for the multiplicity of tests carried out).

In univariate analysis, children who remained free of malaria had significantly higher levels of IgG1antibodies, and lower levels of IgG2 antibodies to total *P.falciparum* extract than children who had one or several malaria attacks (not shown). Similarly, children who remained free of malaria had higher levels of antibodies against Glurp-R0, SERP and the 3 MSP3-derived antigens than children of the same mean age who experienced malaria attacks. In an univariate type of analysis (Figure 1), several IgG sub-classes to those three antigens were found potentially associated with a reduced risk of clinical malaria during the follow-up period: anti-Glurp-R0, anti-SERP and anti-MSP3b IgG1, and IgG3 responses were higher among children free of malaria. Anti-DG210 IgG1 were also high in children with no clinical attack whereas IgG4 against DG210 and MSP3CT were higher in children with malaria attacks. In contrast, there was a trend for IgG1 and IgG3 anti-AMA1 levels to be higher in children with malaria attacks compared to children without clinical malaria attacks.

The potential association between these various antibody responses and the relative susceptibility to malaria attacks over the 51 months of follow-up was then tested by multivariate analysis. The variable that we wished to explain, i.e. the number of malaria attacks, was tested by taking into account several potentially explanatory variables including age, together with antibody responses used as continuous variables. Protection from malaria attacks was found significantly associated with high anti-MSP3b IgG1 responses [L-R Chisquare = 5.99; *P* = 0.0143], and independently, with high anti-MSP3b IgG3 responses [L-R Chisquare = 5.33; *P* = 0.0208]. The indications obtained with MSP3b peptide were confirmed by the use of additional antigens corresponding to either the C-terminus of the molecule (MSP3-CT) or to the conserved region of MSP3 (DG210), targeted by biologically active antibodies. Protection from malaria attacks was also found associated with high anti-DG210 IgG1 levels (L-R Chisquare = 8.1; *P* = 0.004), high anti-DG210 IgG3 (L-R Chisquare = 5.1; *P* = 0.027) and low anti-DG210 IgG4 levels (L-R Chisquare = 7.4; *P* = 0.006), as well as with high anti-MSP3-CT IgG1 responses (L-R Chisquare = 6,1; *P* = 0,013), high anti-MSP3-CT IgG3 responses (L-R Chisquare = 4.9; *P* = 0.027), and with low anti-MSP3CT IgG4 responses (L-R Chisquare = 4.4; *P* = 0.035). Anti-MSP3-CT IgE responses were also tested and found similar in children with (1.24±0.43) and in children without malaria attacks (1.11±0.10; *P* = 0.286). No other antibody specificity showed a similar association with protection in Mlomp, as immune responses to AMA1, Glurp-R0 and Serp became no longer significant in the more stringent multivariate analysis.

### Helminthic Infections Significantly Affect the Isotype Balance of Anti-MSP3 Antibodies

**Figure 2:**
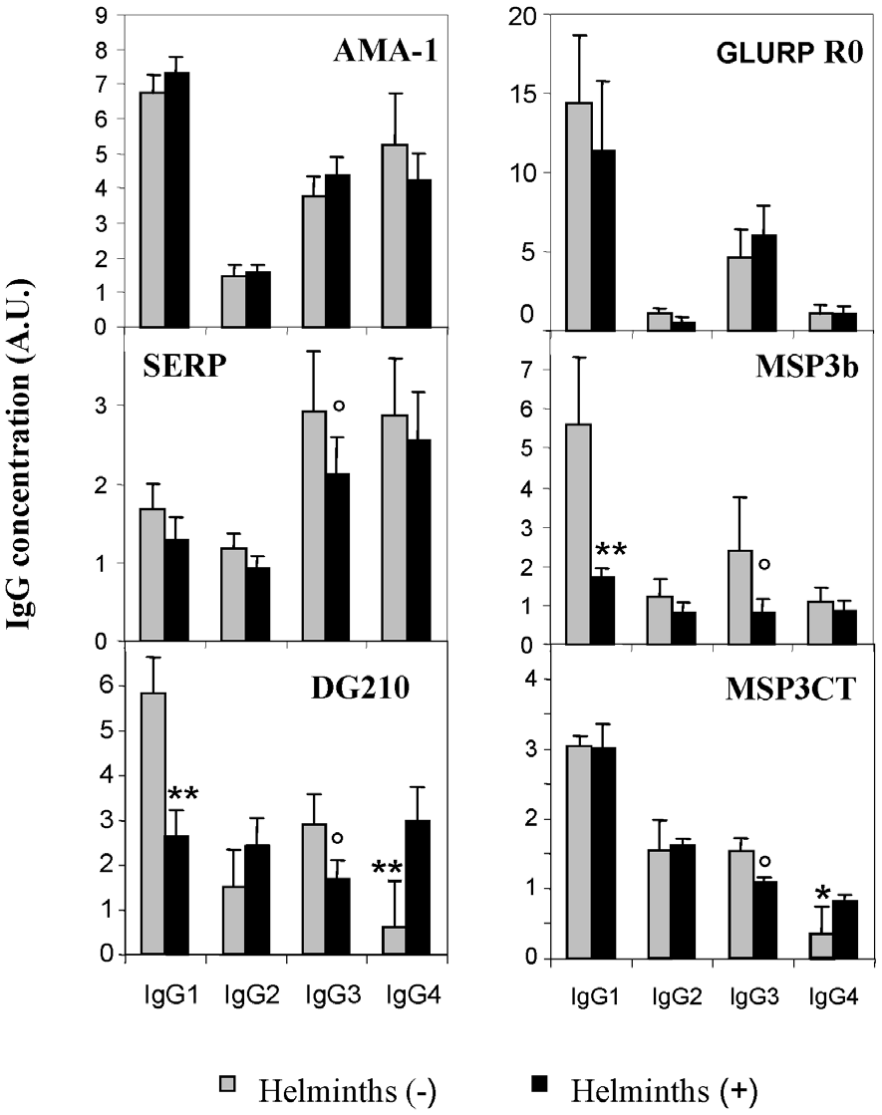
Anti-MSP3 cytophilic Antibody responses are significantly lower in worm-positive than in worm-free children. Mean reactivity and standard errors of the mean IgG A.U. obtained in ELISA assays with sera from children harbouring (helminths +), or not (helminths −) helminthic infection(s) using different antigens including AMA-1, SERP-SERA, GLURP-R0, MSP3b, DG210 and MSP3CT. Labels indicate when differences were found significant in univariate analysis: ° when 0.06>p≥0.05, * when 0.05<p≥0.0021 and ** when p<0.0021 (a p value of 0.0021 accounts for the multiplicity of tests carried out).

As shown in Figure 2, several antigen-specific isotype responses were found to be lower in children carrying worms as compared to children free of intestinal helminth infections. In univariate analysis, this was the case for anti-Glurp-R0 IgG1, anti-SERP IgG3 (*P* = 0.056), anti-MSP3b IgG1 (*P* = 0.0013), anti-MSP3b IgG3 (*P* = 0.0581), anti-DG210 IgG1 (*P* = 0.0126), anti-DG210 IgG3 (*P* = 0.057), anti-MSP3CT IgG3 (*P* = 0.053). IgG4 isotype responses to DG210 (*P* = 0.018) and to MSP3CT (*P* = 0.026) followed an opposite pattern, being found at higher levels in children carrying worms. In contrast, no significant change in the pattern of anti-AMA1 antibody responses was found associated with the presence or absence of intestinal helminths.

However, in multivariate analysis, the presence of intestinal helminths was found to be associated with significantly lower levels of anti-MSP3b IgG1 (L-R Chisquare = 5.27; *P* = 0.013); lower levels of anti-MSP3b IgG3 (L-R Chisquare = 4.72; *P* = 0.029), and conversely, with higher levels of anti-MSP3b IgG4 antibodies (L-R Chisquare = 6.83; *P* = 0.009). This relationship was also found when antibodies to DG210 and MSP3CT were tested in similar conditions. The presence of intestinal helminths was found associated with lower levels of anti-DG210 IgG1 (L-R Chisquare = 6.77; *P* = 0.0093) and higher levels of anti-DG210 IgG4 (L-R Chisquare = 6.99; *P* = 0.0082). When intestinal helminths were present, lower levels of anti-MSP3CT IgG1 (L-R Chisquare = 4.07; *P* = 0.043), lower levels of anti-MSP3CT IgG3 (L-R Chisquare = 8.27; *P* = 0.004) and higher levels of anti-MSP3CT IgG4 (L-R Chisquare = 7.61; *P* = 0.006) were detected. Of note the changes in IgG4 responses were highly significant. The pattern of immune responses identified in helminth positive children, as compared to helminth negative children, is coherent with an increased Thelper 2 type of immune response associated with helminthic infection.

We also investigated anti-MSP3 IgE responses so as to determine if there existed an association between helminth infections and the *P. falciparum*-specific IgE levels. No difference between worm carriers and worm free children was detectable and the mean IgE arbitrary Unit was 1.05±0.371.

Thus, in total, high MSP3-specific cytophilic responses were found associated both with a limited number of malaria attacks and with the absence of intestinal helminths.

## Discussion

In the present study we found a statistically significant correlation between three elements, namely: i) worm carriage, ii) a decrease in cytophilic IgG1 and IgG3 antimalarial response together with an increase in non cytophilic IgG4, and iii) an increased occurrence of malarial attacks.

Results obtained in Casamance confirm on the one hand, the association with protection of anti-MSP3 cytophilic responses [1] and on the other hand, confirm in one additional setting, that worms augment malaria morbidity [2]. However, in addition to previous case/control studies showing a worm-related increase of clinical malaria, the present study supports for the first time the initial immunological hypothesis that this clinical outcome is indeed related to a Th2 worm-driven effect upon anti-plasmodial responses.

Out of the different antigens studied, the association between clinical protection and cytophilic responses was found in univariate analysis for 3 of them, MSP3, Glurp and Serp/Sera, three molecules known to trigger the Monocyte-dependant mechanism, though not for the 4^th^ antigen, AMA-1 which is considered to act by antibodies inhibiting merozoite invasion. However, as has been observed in other settings, the association with MSP3 responses was the strongest and ultimately proved to be the only one remaining significantly associated with protection in multivariate analysis. This was confirmed using 3 different constructs derived from MSP3. As observed previously, the IgG3 isotype of MSP3 antibodies was associated with clinical protection [1], [29]. In contrast with previous findings, the anti-MSP3b IgG1 response in Mlomp children was also significantly associated with protection, this being either an age-related and/or a transmission-related phenomenon. It is reminiscent of the dominant IgG1 response observed in MSP3 immunized European volunteers, and could be related to the lower transmission conditions prevailing in Mlomp as compared to previous studies performed in Dielmo for instance (i.e. IgG1 might constitute the initial antibody response which would be superseded by IgG3 under higher antigen stimulation conditions).

In multivariate analysis, helminthic infection was found associated with an imbalance in favor of non-cytophilic antibodies directed to the molecules mediating ADCI, but not to that mediating merozoite invasion-inhibition. Here as well, and in agreement with the association with protection, the worm-induced imbalance was significant in multivariate analysis only for anti-MSP3 antibodies. In this case, both the cytophilic IgG1 and IgG3 responses were decreased, whereas the non-cytophilic IgG4, which can compete with the former two was significantly increased, relative to IgG1 and IgG3 levels. This was also consistent using 3 MSP3 constructs. These results indirectly support the importance of a Fcγ trigerred monocyte effect *in vivo* in exposed individuals. They also, once more, stress the potential relevance of antibodies to MSP3, as compared to other ADCI targets, and other vaccine candidates, a finding consistent with previous studies in other African and Asian settings [1], [29].

Among possible confounding factors, environmental factors can be likely excluded as in most families, there were both helminth free and helminth-infected children suggesting a similar exposure in the household and immediate surroundings. Conversely, the present observational study does not allow to formally conclude to a direct causal relationship between hookworm carriage, imbalance towards non-cytophilic antimalarial response and occurrence of malarial attacks, and does not exclude that genetic factors, for instance, could also contribute to either one of those 3 parameters.

However, the hypothesis of a causal relation is supported by observations made in other fields than malaria. The influence of worms upon non-malaria specific immune responses in human is actually well documented [31], [32], [33], [34]. For instance, the deleterious effect of worms is documented in the field of responses to currently employed vaccines, e.g. those used in the extended program of immunisation. It is well known that worms can interfere or impede a proper vaccine-induced sero-conversion, and vaccine induced protection, particularly for those vaccines such as tetanus toxoid, tuberculosis, cholera toxin in which protection relies on Th1 type of responses [31], [35], [36], [37], [38]. Helminth infections are known to drive the immune system towards Th2 type of responses whereas protection against malaria is associated with Th1 type of immune responses [20], [39], [40], [41]. Helminth infections are also known to induce marked and highly polarized immune responses characterized by increased Th2 cytokine production and down-regulated Th1 type immune responses (19,30,35,40–42).

Findings in rodents, in which similar quantitative and qualitative immune responses changes were detected, are convergent. Higher parasite loads were reported in helminth-infected animals [20], increased severity of malaria infections (a non lethal *P. yoelii* strain becomes lethal), alteration of innate responses, and a profound decrease in Th1 responses [22]. Helminthic infections have also been found associated with a major increase in T-regulatory cells activity, and a modulation of inflammatory responses [42], [43]. Of note, one study did not illustrate a detectable impact of worm carriage on a rodent model of cerebral malaria, suggesting that various experimental host parasite combinations can yield different results [23]. A recent immuno-epidemiological study brings support to our findings [44]. Though it did not adress the susceptibility to disease, nor that of antibody responses, it indicated deep modifications of cellular responses to a *P.falciparum* extract in wormy individuals, leading to a contra-inflammatory profile of responses. These results confirm in humans our findings in rodents [22] that worms induce a T-regulatory cell activity with a reduction of pro-inflammatory type of cytokines, which are required to generate Th1 responses [44].

Our observations that intestinal worms can affect the quantity and quality of antimalaria immune responses have implications for malaria control (treating worm infections could achieve a faster acquisition of protection against malaria) [2] and for the design of future malaria vaccine phase II-b efficacy trials, with current malaria vaccine candidates (worms could be a major confounding factor, that might deserve to be controlled for in a rigorous manner).

At least, the detection of every category of helminths, including a quantification of worms loads, should now be part of the biological investigations performed during malaria surveys or phase IIb vaccine trials and be included in the final bio-statistical model aimed at interpreting results.

We have recently reported, in the village of Dielmo [1] that a subset of ca. 20% of young children readily developing cytophilic classes of IgG, was protected early in life against malaria, whereas the majority, developing predominantly non cytophilic responses, were prone to repeated malaria attacks until they switched to cytophilic classes. An attractive hypothesis is that the first group may differ from the second one by low or absence of worm infections, and/or associated genetic factors.

Even if this study substantiates the immunological role of worms in the isotype imbalance seen in the majority of children, it is likely that it is not the only influence. Several other factors may potentially contribute to the outcome; for instance, children are more prone to produce, early in life, IgM non-cytophilic classes of antibodies in contrast to adults who produce both IgG2 and IgM non-cytophilic responses. It is also plausible that there are genetic factors at the origin of a preferential switch towards a particular class or subclass of antibody. A detailed analysis of the underlying factors would require large cohort studies as there will be a need to combine an analysis of the genetic background of human populations, of parasite populations, and of the almost unlimited combinations of concomitant infections by parasites, viruses or bacteria, that are highly prevalent under tropical conditions [17], [45], [46]. The resulting multi-factorial equation is obviously complex to analyze and this is likely the main factor that has delayed until now the identification of the worm effects upon malaria.

Our study and the underlying hypothesis bring new elements contributing to improving our understanding of the dynamics of *P.falciparum*/human immune interaction and for the very long delay required to acquire protective immunity against malaria related to a dominance of non-cytophilic responses, the latter being at least in part now attributable to worm carriage
